# Immature to total neutrophil ratio as an early indicator of early neonatal sepsis

**DOI:** 10.12669/pjms.35.1.99

**Published:** 2019

**Authors:** Erum Saboohi, Farhan Saeed, Rashid Naseem Khan, Muhammad Athar Khan

**Affiliations:** 1 Dr. Erum Saboohi, MBBS, MD. Senior Registrar, Department of pediatrics Liaquat College of Medicine & Dentistry, Karachi, Pakistan; 2 Dr. Farhan Saeed, MBBS, DCH, FCPS. Assistant Professor, Department of Pediatrics Liaquat College of Medicine & Dentistry, Karachi, Pakistan; 3 Dr. Rashid Naseem Khan, MBBS, MD, MCPS. Principal (Medical), Liaquat College of Medicine & Dentistry, Karachi, Pakistan; 4 Dr. Muhammad Athar Khan, MBBS, MCPS, MBA, DPH, DCPS-HPE, DCPS-HCSM, PGD-Statistics, CCRP. Associate Professor, Department of Community Medicine, Liaquat College of Medicine & Dentistry, Karachi, Pakistan

**Keywords:** Neonatal Sepsis, Early onset sepsis (EOS), CRP, I/T ratio, Blood culture

## Abstract

**Background & Objectives:**

Neonatal septicemia is responsible for 1.5 to 2.0 million deaths/year in the under developed countries of the world. Pakistan is number three among these countries and accounts for 7% of global neonatal deaths. The objective of the study was to determine the role of simple hematological parameter, immature to total neutrophil ratio (I/T ratio) in diagnosing early onset neonatal bacterial infection.

**Methods:**

A descriptive cross-sectional study was conducted in Neonatal Intensive Care Unit of Liaquat College of Medicine & Dentistry (LCMD) Hospital from January 2016 to January 2017. A total 85 neonates were admitted with clinical suspicion of presumed early onset sepsis or who had potential risk factors for sepsis like prematurity, prolonged rupture of membranes was carried out. After taking informed consent from parents of admitted neonates, data was collected in a structured questionnaire. Laboratory workup included White blood cell count, CRP, absolute neutrophil count, immature neutrophil count while blood C/S was kept as gold standard. Empirical antibiotics started after sample collection for workup. Manual differential count and immature neutrophil count of the peripheral blood smear was performed by a senior technician masked to clinical information. I/T ratio was calculated from WBC, neutrophils and immature neutrophil count by a simple formula.

**Results:**

Out of 85 neonates, 13 had positive blood cultures (15.29%). The mean white blood count was 18761.18 ± 8570.75 and mean I/T ratio was 0.1622 ± 0.0419. About 50% of proven sepsis cases had WBC higher than 26000 as compared to 50% of cases for negative diagnoses that had WBC <15500. The mean I/T in positive CRP 0.204 ± 0.04 was non-significantly higher as compared to negative CRP 0.151 ± 0.034 (p =0.084). Point biserial correlation revealed that I/T ratio was significant strong correlation (r_pb =_ 0.721, p < 0.001) and overall I/T ratio was a good indicator of a positive and negative blood culture result. The sensitivity, specificity, positive predictive value (PPV) and negative predictive value (NPV) of I/T ratio were 76.47%, 83.82%, 54.16% and 93.44% respectively. Similarly majority of neonates having high I/T ratio also depicts positive C-reactive protein (CRP) (NPV 91.23%). Therefore, both I/T and CRP showed a high negative predictive value (I/T = 93.44% and CRP = 91.23%) in this study.

**Conclusion:**

I/T ratio is a useful tool for early onset sepsis (EOS) with reasonable specificity but cannot be relied upon as sole indicator. Combination of normal immature to total neutrophil Ratio with negative CRP values in neonates with presumed sepsis is an indicator of non-infected neonate which comprised 78.8% of our study population.

## INTRODUCTION

Neonatal sepsis is described as a suspected or demonstrated infection in newborn, a systemic inflammatory response syndrome with variable sign and symptoms caused by pathogens with or without accompanying bacteremia.^1^ The incidence of Neonatal sepsis is approximately 8 per 1000 live births and as high as 13 to 27 per 1000 for newborns weighing < 1500 gms.^2^ Neonatal septicemia is responsible for 1.5 to 2.0 million deaths/year or between 4000 to 5000 deaths/day in the underneath advanced countries of the world.^3^ Two thirds of the world’s neonatal deaths occur in just 10 countries, frequently in Asia. Pakistan accounts for 7% of global neonatal deaths. The predominant causes are infections (36%), preterm births (28%) and birth asphyxia (23%) accounting for about 87% of neonatal deaths worldwide.^4^

Risk factors for neonatal sepsis are low birth weight infants, birth asphyxia, respiratory compromise at birth, maternal risk factors and congenital anomalies.^5^ Diagnostic tools for identification of EOS includes prenatal screening of high risk mothers to clinical and laboratory identification of newborns with presumed sepsis. Various diagnostic tools have been extensively studied over many years for EOS like WBC, BANDS, ANC, immature to total neutrophil ratio, CRP, interleukin six, procalcitonin.

Blood culture remains a gold standard for diagnosing neonatal sepsis but results are typically obtained after three to five days and its accuracy varies between eight and 73% in various studies.^6^ However, there are some screening tests (WBC, Platelets, Micro Erythrocyte sedimentation rate(ESR), Absolute Neutrophilic Count (ANC), C-Reactive Protein (CRP), (I/T) ratio, nitroblue teterazolium (NBT), serial Interleukin-6 (IL-6) and pro-calcitonin) that could predict sepsis within 6 to 8 hours.^7^ The ANC(<1000/ul) and the I/T ratio(≥ 0.2)give the clue about the early-onset sepsis in newborns.^8^ Rapid diagnostic tests like CRP, WBC indices may be used as a screening approach for early diagnosis and treatment of sepsis.^9^

In a prospective study by Kredit T et al in 185 neonates, showed a high NPV of CRP and I/T ratio in early and late onset infection (90% to 98%).^10^ The sensitivity of I/T ratio has ranged from 60 to 90%. Therefore, when diagnosing neonatal sepsis, the elevated I/T ratio values should be considered in conjunction with other clinical signs.^11^ From previous studies it was concluded that I/T ratio of >0.80 fairly approximates Neutrophils Storage Pool in bone marrow thereby saving the neonates from bone marrow examinations. This ratio is quiet helpful in identifying newborns who might be benefitted by G-CSF (granulocyte colony stimulating factors).^12^

Early diagnose of EOS by simple hematological ratio can improve clinical outcome in these neonates.^13^,^14^ The significance of this study is to test the diagnostic accuracy of I/T ratio for EOS before the results of blood culture and sensitivity are available, and from routine CBC sample collection, important hematological marker I/T ratio can be determined. Reasonable clinical judgment with I/T Ratio provides rational basis for treatment decision in neonatal sepsis. Such strategy significantly reduces unnecessary antimicrobial therapies which lengthen hospital stay and can otherwise permit emergence of resistant pathogen strains.

## METHODS

This cross sectional study was conducted to identify and evaluate a scheme for an early detection of neonatal sepsis. Data of 85 infants admitted with presumed sepsis in the NICU of the Liaquat College of Medicine & Dentistry (LCMD) Hospital, Karachi, from January 2016 to January 2017 was collected and analyzed.

Presumed sepsis was defined if the blood culture was negative but there was a strong clinical suspicion for infection. Neonates with positive blood culture were defined as having confirmed sepsis. The sample size of the study was calculated by WHO sample size calculator^15^ taking sensitivity 75%, specificity 95%, expected prevalence 50%, desired precision 10.5% and confidence level 95%, the calculated sample size was (n = 85).

Leucocyte counts were done on coulter counter in lab. Differential and immature neutrophil counts were performed manually after making suitable thin film smears by senior Lab technician. CRP levels considered negative when < 5 mg/dl. Blood cultures collected with proper a-septic techniques. Liquid thioglycolate lisp(oxoid) was used for blood culture and 2 ml of neonatal blood was added to 20 ml of medium. Inoculated culture bottles were incubated at 36°C for seven days and examined for growth every 24 hours; negative culture reports given after 7 days. Blood culture taken as gold standard. Tests used for screening were

1-total leukocyte counts either less than 5000 or more than 20000/cumm
2-neutropenia/ neutrophilia (age adjusted count, described by Monroe et al 1979)
3-immature to total neutrophilic count ratio (I/T ratio > 0.2)
4-positive C- reactive protein

Immature to total neutrophil ratio (I/T ratio) calculated by the following formula:


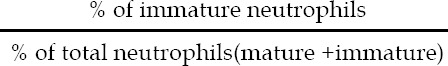


Statistical analyses were performed using SPSS software version 22 for Windows. Continuous variables like I/T ratio, age, weight, ANC and WBCs were presented as mean and standard deviation, while categorical variables like gender, and distribution of immature Neutrophils were presented as frequencies and percentages. Independent sample t-test was applied to find out the difference among blood culture and study parameters like I/T ratio, age, weight and WBCs. Point biserial correlation was calculated to evaluate the relationship among different study predictors. Chi-square test / Fisher’s exact was applied to determine the association of blood culture with I/T ratio and CRP. A two-sided p value <0.05 was considered significant.

The current study was approved by the ethical committee of LCMD hospital Karachi. After taking informed consent from parents of admitted neonates, data was collected in a structured questionnaire.

## RESULTS

A total 85 neonates were registered after screening, out of which 48 (56.5%) were males and 37 (43.5%) were females. The mean age and weight of the neonates were 1.58 ± 0.76 days and 2.38 ± 0.55 kg (maximum 4 kg and minimum 1.1 kg) respectively.

The mean white blood count was 18761.18 ± 8570.75 and mean I/T ratio was 0.1622 ± 0.0419. ANC ranged between 3100-15000/ul. None of the patient showed neutropenia. The average neutrophil count was 64.88 ± 10.12 while immature neutrophil was 10.66 ± 2.74. In this study frequency analysis was also performed for WBC count for positive and negative sepsis cases. There was only one case with WBC count <5000/cumm with proven sepsis. Culture proven sepsis occurred in 15 neonates accounting for 17.64% of cases. WBC count was abnormal in 10 of 15 culture proven cases (66.66% specificity). Elevated I/T ratio (>0.2) identified in 7 of 15 culture proven cases (46.66% sensitivity). 55 out of 70 culture negative cases had normal I/T ratio (78.57% specificity).

Frequency analysis is performed for WBC count for the positive and negative sepsis cases. There is only one case with < 5000 cumm with proven sepsis. About 50% of positive cases have WBC higher than 26000 as compared to 50% of cases for negative diagnoses that have WBC less than 15500/cumm. WBC, together with higher I \ T Ratio, could be good indicator for positive diagnosis.

Furthermore, the difference of mean positive blood culture was significantly higher in I/T ratio, age and WBCs as compared negative blood culture. Although, the mean weight with positive blood culture 2.412 ± 0.618 kg was slightly more as compared to negative BC 2.382 ± 0.547 kg but it was statistically non-significant. The mean I/T in positive CRP 0.204 ± 0.04 was non-significantly higher as compared to negative CRP 0.151 ± 0.034 (p =0.084).

Point biserial correlation revealed that I/T ratio was significantly correlated with blood CS (r_pb =_ 0.721, p < 0.001) and it was a good indicator of a positive and negative blood culture result (Table-I).

**Table-I:** Correlation of blood culture with I/T ratio, age, weight and total WBC count.

Parameters	r_pb_ (p-value)	Blood Culture		p-value

		Negative (n=68)	Positive (n=17)	
I/T Ratio	0.721 (< 0.001)*	0.151 ± 0.034	0.204 ± 0.044	< 0.001*
Age (days)	0.280 (0.010)*	1.471 ± 0.722	2.011 ± 0.791	0.010*
Wight (Kg)	0.021 (0.847)	2.382 ± 0.547	2.412 ± 0.618	0.847
WBC	0.488 (< 0.001)*	16683.831 ± 7039.878	27070 ± 9296.220	< 0.001*

WBC: white blood cell; r_bp_: Point biserial correlation was calculated for blood culture with all parameters. Continuous variables were expressed as Mean and Standard deviation and Independent sample t-test was applied.

* Significant p-value.

We also observed that the frequency of CRP negative was significantly lower in the patients of positive blood culture (n = 5, 29.4%) whereas CRP (+ve) in positive blood culture was (n = 16, 23.5%) (p < 0.001).

A difference in proportion of I/T ratio ≥ 0.2 was statistically significant higher in positive blood culture (n = 13, 76.5% v/s n = 11, 16.2% and p < 0.001) in (Table-II). The diagnostic accuracy of I/T ratio revealed that sensitivity, specificity, PPV and NPV in neonatal sepsis were 76.47%, 83.82%, 54.16% and 93.44% respectively (Table-III).

**Table-II:** Association of Blood Culture with I/T ratio and CRP.

Parameters	Blood Culture		p-value

	Negative (n=68)	Positive (n=17)	
***I/T Ratio***			
≥ 0.2	11 (16.2%)	13 (76.5%)	< 0.001*
< 0.2	57 (83.8%)	4 (23.5%)
***CRP***			
-VE	52 (76.5%)	5 (29.4%)	< 0.001*
+VE	16 (23.5%)	12 (70.6%)	

Categorical variables were expressed as Frequency and Percentage.

* Significant p-value calculated by Fisher Exact / Chi-square test.

**Table-III:** Diagnostic Accuracy of I/T ratio in cases with EONS.

	Proven Sepsis (CS Positive)	Presumed Sepsis (CS Negative)
I/T ratio > 0.2	TP(true positive) = 13	FP(false positive) = 11
I/T ratio < 0.2	FN(false negative) = 4	TN(true negative) = 57

Specificity = 83.82%, Sensitivity = 76.47%, PPV = 54.16%, NPV = 93.44%

## DISCUSSION

Total 85 neonates with presumed early onset neonatal sepsis (EONS) based on history and examination were investigated. 48 out of 85 admitted neonates with clinical suspicion of early onset sepsis were low birth weight i-e < 2.5 kg accounting for 56.4% cases Out of 48 LBW neonates, 30 (62.5%) were preterm (<37 weeks gestation), a finding that was consistent with similar previous studies on neonatal sepsis.^16^,^17^ There was an inverse relationship of birth weight to infection rate due to impaired cellular immunity in low birth weight neonates which make them more susceptible to acquire infections.^18^,^19^

About 50% of positive cases have WBC count >26000/cumm as compared to 50% of cases with negative diagnosis that have WBC count <15500/cumm. Studies have shown that screening WBC count alone are poor predictor of neonatal infection^20^ as some non-infectious conditions also cause neutrophilia like birth asphyxia and stress. CRP showed specificity of 76.47%, sensitivity of 70.58%, PPV of 42.85% and NPV of 91.23%.

I/T ratio reference ranges are obtained from Schmutz chart.^21^ A ratio of ≥0.2 is highly sensitive marker of neonatal septicemia.^22^ In the present study I/T ratio of ≥0.2 showed specificity of 83.8%, sensitivity of 76.5%, PPV of 54.6% and NPV of 93.44%. Hence, I/T ratio of ≥0.2 our results are comparable to other studies^22^. Some previous studies have shown different results in this parameter which may be due to variation in blood sampling, severity of sepsis, age of patient and investigative criteria followed, sensitivity: 90% to 100%, specificity: 30% to 78%, PPV: 11% to 51% and NPV: 99% to 100%.^23^

The present study revealed that, from the various sepsis screen parameters, the I/T ratio showed high specificity and high negative predictive value. The result from this study depict the role of I/T ratio as an indicator to exclude early onset neonatal sepsis as negative predictive value is on higher side. This is in agreement with the previous studies which displayed NPV in the range of 90%to 98% for I/T ratio ≥0.2 in excluding EOS. Similar study with a NPV of 100% of I/T ratio >0.2 conducted by Murphy et al in 2012 and others.^24^,^25^

Furthermore, the present study revealed that even simple hematological ratio can help in ruling in or ruling out EOS, thereby decreasing hospital stay, alleviating the anxiety of parents and help preventing development of antibiotic resistant pathogenic strains. This is especially important for the various resources limited clinical setups in underdeveloped countries.

The significance of the study is that it represents a thorough statistical analysis of I \ T Ratio as an early indicator of EONS. This could allow practitioners to take preventive measures at an earlier level. Analysis of other parameters that could contribute to early diagnosis can be advantageous. The statistical significance of other parameters, for example, CRP, preterm births and weights need to be further investigated to establish whether these can improve accuracy of EOS diagnosis when considered together with I \ T Ratio.

### Limitations of the study

We had only few infants with positive blood culture. Another important issue when conducting research on sepsis in neonates is that there is no perfect gold standard with which to compare the results with. When calculating sensitivity and specificity the new result is compared with blood culture, a method with widely known limitations in both sensitivity and specificity. Another limitation is that the study included material from a single tertiary care centre; this increases the risk that the results only reflect the local situation and this must be taken into consideration before the results are generalized to other locations.

## CONCLUSION

Though there are several markers to diagnose neonatal sepsis, the search for the ideal marker is still on. In the present study I \ T Ratio showed high specificity and high negative predictive value for neonatal sepsis. The predictive value of elevated I \ T Ratio and simplicity of the test justifies its routine use in early diagnosis of early onset neonatal sepsis. The markers from the present study and the interpretation from chosen cutoff values ought to be tested in a larger prospective clinical study. Future research in the area of diagnosing neonatal sepsis is highly necessary for the benefit of health care professionals dealing with infected neonates and their attendants.

I/T ratio estimation does have a role in diagnosing early neonatal septicemia but it is not sensitive enough to be relied upon as the sole indicator. When this readily calculated ratio is used together with clinical signs of EONS, a negative test result may help in ruling out EONS. Based on the results of this study, it may be concluded that the early discontinuation of antibiotics (within 24 to 48 hours) in neonates with suspicion of EONS can be planned on I \ T Ratio results in conjunction with improving clinical signs. Its usefulness may be enhanced when it is considered in conjunction with other sepsis screen markers like CRP and WBC in ruling out sepsis. More data should be available to draw the final decision. However, we suggest designing a scoring system for the screening of EONS by using combination of tests.
